# Genome-wide expression profiles of subchondral bone in osteoarthritis

**DOI:** 10.1186/ar4380

**Published:** 2013-11-15

**Authors:** Ching-Heng Chou, Chia-Chun Wu, I-Wen Song, Hui-Ping Chuang, Liang-Suei Lu, Jen-Huei Chang, San-Yuan Kuo, Chian-Her Lee, Jer-Yuarn Wu, Yuan-Tsong Chen, Virginia Byers Kraus, Ming Ta Michael Lee

**Affiliations:** 1 Institute of Biomedical Sciences, Academia Sinica, 128, Sec. 2, Academia Road, Nankang District, Taipei 11529, Taiwan; 2 National Center for Genome Medicine, Institute of Biomedical Sciences, Academia Sinica, 128, Sec. 2, Academia Road, Nankang District, Taipei 11529, Taiwan; 3 Department of Pathology, Duke University School of Medicine, DUMC 3712, Durham, NC 27710, USA; 4 Department of Orthopaedic Surgery, Tri-Service General Hospital, National Defense Medical Center, 325, Sec. 2, Cheng-gung Rd., Neihu Dist., Taipei 11472, Taiwan; 5 Graduate Institute of Life Sciences, Tri-Service General Hospital, National Defense Medical Center, 114 No.161, Sec. 6, Minquan E. Rd., Neihu Dist., Taipei 114, Taiwan; 6 Translational Resource Center for Genomic Medicine, Institute of Biomedical Sciences, Academia Sinica, 128, Sec. 2, Academia Rd., Nankang District, Taipei 11529, Taiwan; 7 Department of Orthopedics, School of Medicine, College of Medicine, Taipei Medical University, 250 Wu-Hsing Street, Taipei 110, Taiwan; 8 Department of Orthopedics, Taipei Medical University Hospital, 250 Wu-Hsing Street, Taipei 110, Taiwan; 9 Department of Pediatrics, Duke University School of Medicine, DUMC 3352, Durham, NC 27710, USA; 10 Department of Medicine, Duke University School of Medicine, 2301 Erwin Road, Room 1102, Durham, NC 27710, USA; 11 Graduate Institute of Chinese Medical Science, China Medical University, No.91 Hsueh-Shih Road, Taichung 40402, Taiwan; 12 Laboratory for International Alliance on Genomic Research, RIKEN Center for Integrative Medical Sciences, 1-7-22 Suehiro-cho, Tsurumi-ku, Yokohama, Kanagawa 230-0045, Japan

## Abstract

**Introduction:**

The aim of this study was to evaluate, for the first time, the differences in gene expression profiles of normal and osteoarthritic (OA) subchondral bone in human subjects.

**Methods:**

Following histological assessment of the integrity of overlying cartilage and the severity of bone abnormality by micro-computed tomography, we isolated total RNA from regions of interest from human OA (n = 20) and non-OA (n = 5) knee lateral tibial (LT) and medial tibial (MT) plateaus. A whole-genome profiling study was performed on an Agilent microarray platform and analyzed using Agilent GeneSpring GX11.5. Confirmatory quantitative reverse-transcription polymerase chain reaction (qRT-PCR) analysis was performed on samples from 9 OA individuals to confirm differential expression of 85 genes identified by microarray. Ingenuity Pathway Analysis (IPA) was used to investigate canonical pathways and immunohistochemical staining was performed to validate protein expression levels in samples.

**Results:**

A total of 972 differentially expressed genes were identified (fold change ≥ ± 2, *P* ≤0.05) between LT (minimal degeneration) and MT (significant degeneration) regions from OA samples; these data implicated 279 canonical pathways in IPA. The qRT-PCR data strongly confirmed the accuracy of microarray results (R^2^ = 0.58, *P* <0.0001). Novel pathways were identified in this study including Periostin (*POSTN*) and Leptin (*LEP*), which are implicated in bone remodeling by osteoblasts.

**Conclusions:**

To the best of our knowledge, this study represents the most comprehensive direct assessment to date of gene expression profiling in OA subchondral bone. This study provides insights that could contribute to the development of new biomarkers and therapeutic strategies for OA.

## Introduction

Osteoarthritis (OA) is the most prevalent musculoskeletal disorder and the most common form of arthritis among older individuals in most countries worldwide; OA constitutes a large economic burden due to the associated costs of medical care and lost wages [1,2]. Although degeneration of cartilage is the major characteristic of OA, the disease also involves the entire joint organ, including structural modifications of underlying subchondral bone, pathological changes of the meniscus and synovitis [3,4]. Maintenance of normal joint structure and function relies on load adaptation of the cartilage and bone. Disruption of the physiological relationship between these tissues contributes to the development of OA pathology [5].

Increasing evidence indicates that the subchondral bone, consisting of the subchondral plate and subchondral spongiosa, plays a major role in the initiation and progression of OA [6]. Magnetic resonance imaging-based visualization of the whole knee structure demonstrates that increased tibial subchondral bone volume is associated with severity of knee OA [7]. Kraus and colleagues demonstrated that subchondral bone texture can be used as a biomarker to predict progression of knee OA [8]. A study comparing two guinea pig strains indicates that an increasing rate of knee subchondral bone remodeling is associated with the process of cartilage deterioration [9]. Moreover, human bone cell culture studies have shown that factors released from bone cells might affect cartilage metabolism [10,11]. These studies provide insights into a temporal relationship between subchondral structural changes and alterations in articular cartilage during the development of OA; they also underscore the importance of elucidating the molecular changes in human subchondral bone to improve our understanding of the pathogenesis of OA.

Whole-genome microarray is a common technology for studying the behavior of many genes simultaneously. All of the gene expression microarray profiling studies in OA so far have been performed on human articular cartilage, meniscus or synovium; however, none have been performed on human subchondral bone tissue directly. Only one study has reported the gene expression profiles of subchondral bone in an early OA experimental mouse model [12]. A few studies have evaluated the gene expression profiles of distal trabecular bone from human OA [13,14], but this site is remote from subchondral bone rather than a reflection of alterations locally in the OA joint. This paucity of subchondral bone microarray studies is most probably due to the difficulties associated with isolation of high-quality RNA from subchondral bone. As described in our previous study [15], we have developed a method of precisely harvesting specific regions of subchondral bone tissue and for subsequently isolating high-quality RNA from these specimens. Our methodology has made it possible to perform microarray analyses of human subchondral bone samples.

Our goal in this study was to evaluate the association of subchondral bone gene expression with bone histomorphometry at sites of intact articular cartilage and osteoarthritic lesioned cartilage. To our knowledge, this is the first study to successfully perform microarray analyses of human knee subchondral bone in OA and non-OA samples, thereby providing clues to the pathogenic mechanisms of OA that could inform development of new diagnostic markers and therapeutic targets.

## Methods

### Human knee joint tissues

Human osteoarthritic tibial plateaus with medial compartment dominant OA and macroscopically normal lateral compartments were obtained during total knee joint replacement surgery from knee OA patients. The diagnosis of OA was based on the criteria for knee OA of the American College of Rheumatology [16]. Normal tibial plateaus were obtained at autopsy or within 8 hours after amputation surgery from donors with nonarthritic knee joints.

To ensure consistency of sampling of prespecified regions of interest, the anatomic orientation was indicated on the freshly isolated specimens by marker pen and then all specimens were stored immediately in liquid nitrogen. Methods for precisely obtaining specimens of a particular type from specific regions of interest of the joint have been described previously [15]. The tissues were manipulated at all times under liquid nitrogen to prevent RNA degradation. Regions of interest were harvested for histological analysis of the articular cartilage, and subchondral bone structural parameters were determined by micro-computed tomography; regions immediately adjacent were harvested for RNA isolation and microarray analysis. For microarrays, RNA was isolated from subchondral bone from four regions: the outer and inner lateral tibial (LT) plateau and the inner and central medial tibial (MT) plateau (Additional file 1). The gene expression intensities changed little within the LT plateau (between inner and outer LT regions) and within the MT plateau (inner and central MT regions) (Additional file 2); we therefore based the analyses in the current study on a comparison of gene expression from subchondral bone of 20 outer LT plateaus and 20 MT plateaus from knee OA specimens. These 40 samples were derived from 30 individual knee OA specimens, yielding 10 paired LT and MT samples and 10 unpaired LT and MT samples. The 40 OA samples chosen for microarray analysis represented a subset from our previous study [15], and were chosen to have LT OARSI scores ≤8 and MT OARSI scores ≥18. In addition, five LT and five MT paired subchondral bone samples from non-OA donors were used as controls for the microarray analysis. The characteristics of the samples used for microarray analysis are summarized in Table 1 and Additional file 3.

**Table 1:** Characteristics of the samples used for microarray analysis

	**Osteoarthritis**		**Nonosteoarthritis**	
**Characteristic**	**LT**	**MT**	**LT**	**MT**
OARSI	4.7 ± 1.34	20.8 ± 1.62*	3.00 ± 1.85	3.75 ± 1.67
BV/TV (mm^3^)	21.32 ± 7.47	65.39 ± 12.61*	24.41 ± 3.71	35.26 ± 10.12
SMI	1.56 ± 0.34	−1.69 ± 1.70*	0.99 ± 0.21	0.68 ± 0.73
Tb.Th (mm)	0.18 ± 0.04	0.36 ± 0.09*	0.19 ± 0.01	0.26 ± 0.08
Tb.N (1/mm)	1.16 ± 0.27	1.83 ± 0.33*	1.28 ± 0.21	1.36 ± 0.09
Tb.Sp (mm)	0.51 ± 0.12	0.25 ± 0.09*	0.56 ± 0.09	0.49 ± 0.03
Age (years)	71.10 ± 9.55	69.65 ± 9.55	38.40 ± 13.45	38.40 ± 13.45
Male sex (%)	33	34	36	32
Number	20	20	5	5

Values presented as mean ± standard deviation. BV/TV, percent bone volume; LT, lateral tibial; MT, medial tibial; OARSI, histological scoring system to grade severity of osteoarthritis; SMI, structure model index; Tb.N, trabecular number; Tb.Sp, trabecular separation; Tb.Th, trabecular thickness. *P <0.001 considered significant comparing MT osteoarthritis versus LT osteoarthritis. *P* values were determined by two-tailed unpaired Student’s *t* test.

The study was approved by the institutional review board of Academia Sinica, Tri-Service General Hospital and Taipei Medical University Hospital, and written informed consent was obtained from all of the participants.

### Subchondral bone tissue harvest and RNA isolation

**Figure 1:**
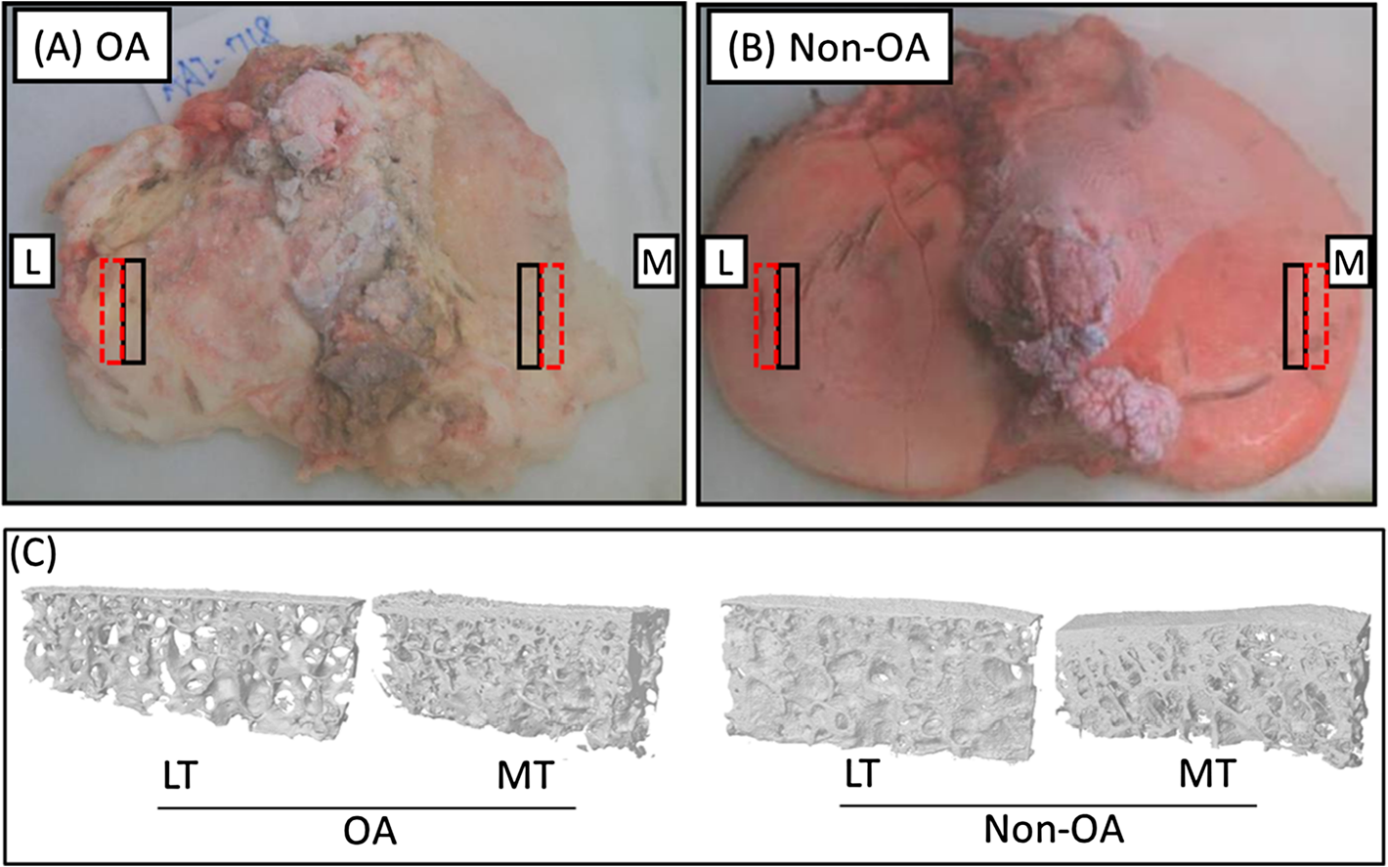
**Sites of micro**-**computed tomography analysis and regions chosen for RNA isolation. (A)** Human osteoarthritic knee tibial plateau (osteoarthritis (OA)) and **(B)** normal knee tibial plateau (non-OA). Red dashed boxes, regions chosen for RNA isolation; black boxes, regions chosen for micro-computed tomography and histological analysis at the two regions of interest. **(C)** Representative three-dimensional images of subchondral bone from the lateral tibial (LT) and medial tibial (MT) plateau from OA and non-OA specimens.

All tissues were sectioned to generate osteochondral slabs 2 to 2.5 mm thick from regions of interest for micro-computed tomography scanning and histological analysis (Figure 1); regions immediately adjacent were isolated for subsequent grinding and RNA isolation using a custom-made workstation as described previously [15]. Approximately 100 mg powdered subchondral bone from the tibial plateau was obtained for RNA extraction. Five milliliters of Trizol (Invitrogen, Carlsbad, CA, USA) were added to 100 mg (dry weight) ground tissue powder and mixed by vortexing until homogeneous. A total of 1 ml chloroform was added to the homogenized sample and centrifuged at 12,000 × *g* for 15 minutes at 4°C to achieve phase separation. The clear aqueous phase was transferred to a fresh tube for a second phase separation with phenol/chloroform/isoamyl alcohol (25:24:1; Sigma-Aldrich, St. Louis, MO, USA), followed by RNA precipitation with pure isopropanol. After washing with 70% ethanol, the air-dried pellet was dissolved in RNase-free water and genomic DNA was removed by DNase digestion (Qiagen, Hilden, Germany) according to the manufacturer’s instructions. To further improve RNA quality, one-half volume of 7.5 M LiCl (Invitrogen) was added and incubated at −20°C for at least 30 minutes followed by RNA precipitation by centrifugation at 12,000 × *g* for 15 minutes. After 70% ethanol washing, the RNA was resuspended in RNase-free water. The RNA concentration and purity were determined by Nano-Drop (NanoDrop Technologies, Wilmington, DE, USA). The RNA integrity number and the 28 s/18 s ratio were estimated using RNA 6000 Nano Assays on an Agilent 2100 Bioanalyzer (Agilent Technologies, Santa Clara, CA, USA) according to the manufacturer’s instructions.

### Microarray analysis

Four hundred nanograms of total RNA per sample were used for one round of cRNA synthesis and amplification. Cyanine 3-labeled cRNA was then purified and hybridized to Agilent whole human genome 44 k microarray chips (Agilent Technologies). All procedures were carried out according to the manufacturer’s instructions (Agilent Technologies, Taipei, Taiwan). The array signal intensities were further analyzed by the Agilent GeneSpring GX software (version 11.5; Agilent Technologies).

Gene expression values of all datasets were normalized using quantile normalization; probes with low signal intensities were excluded by setting the filter above 32. The normalized values were log transformed. Significant differentially expressed genes between lateral and medial samples in OA patients were identified using an adjusted Student’s *t* test (*P* <0.05) corrected for multiple comparisons with the Benjamini–Hochberg test. The results were used to run principal component analysis to uncover the trends among OA and normal samples. Differentially expressed genes between medial and lateral regions among groups were identified by a threshold of ≥2 fold-change and *P* ≤0.05. The hierarchical clustering method, with Euclidean distance and centroid linkage, was further applied to cluster the differences in gene expression levels between samples. Lists of differentially expressed genes were imported into ingenuity pathway analysis (IPA; Ingenuity Systems, Redwood City, CA, USA) to identify functional annotations and biological interactions from the many known relationships between proteins, genes and diseases. The biological interaction scores were defined by the IPA statistical algorithm based on its knowledge base, and the adjusted *P* value was calculated by Fisher’s exact test and corrected for multiple comparisons with the Benjamini–Hochberg test. All microarray raw data are available through the GEO database [GEO:GSE51588].

### Quantitative reverse-transcription polymerase chain reaction validation

To validate results from microarray analysis, we performed reverse-transcription polymerase chain reaction (qRT-PCR) for 85 genes (plus *GAPDH*) on nine additional OA paired LT and MT subchondral bone specimens; eight of these additional OA knee joint specimens have been described previously [15], as well as the qRT-PCR results for some of the genes combined with data yielded from one additional knee OA specimen. Forty-three of these genes were identified for verification on the basis of significant fold-change (≥2) comparing LT and MT microarray results. The remaining 42 genes were identified on the basis of their potential involvement in OA as described previously [15]. The expression of these 42 genes was previously analyzed for seven regions of interest from eight OA cartilage and subchondral bone specimens.

In the current study, we analyzed two regions of interest (LT and MT subchondral bone corresponding to outer LT and center MT of the prior work) in nine specimens (the eight former specimens and an additional OA specimen with available LT and MT regions). The qRT-PCR was performed using the Taqman high-density microfluidic cards (Invitrogen, Carlsbad, CA) according to the manufacturer’s instructions. RNA from each region was converted into cDNA using Superscript III reverse transcriptase (Invitrogen, Carlsbad, CA). The qRT-PCR reactions were performed on the ABI Prism 7900HT Sequence Detection system and the fluorescent signal intensity was analyzed by Sequence Detector v2.3 software (Applied Biosystems, Foster City, California). The levels of cDNA among samples were normalized to the expression of GAPDH and analyzed with the ΔΔCt relative quantification method to identify significant variation in gene expression comparing LT and MT regions in OA samples. Two-tailed unpaired Student *t* tests were performed to evaluate for statistically significant differences in gene expression levels between regions.

### Immunohistochemistry staining

Approximately 2 mm diameter sections from the tibial plateau were fixed in 4% paraformaldehyde (Sigma-Aldrich) overnight and decalcified in 10% ethylenediamine-*N*, *N*, *N*′, *N*′-tetraacetic acid, disodium salt, dehydrate (Sigma-Aldrich) for 2 weeks. After decalcification, the sections were embedded in paraffin and 10 μm sections were prepared. The embedded tissue sections were then deparaffinized, hydrated, and the endogenous peroxidase activity was quenched in 0.3% H_2_O_2_–methanol for 30 minutes. After incubating in 0.5% trypsin/1% CaCl_2_ at 37°C for 30 minutes and blocking with diluted goat serum at 25°C for 1 hour, the sections were incubated with anti-human *PSOTN* (dilution 1:300) antibodies (ab14041; Abcam, Cambridge, MA, USA), and *LEP* (dilution 1:1,000) antibodies (ab16627; Abcam) or nonimmune goat serum at 4°C overnight and reacted with biotinylated secondary antibody for 30 minutes. Signal amplification and staining were performed using Vectastain ABC kits (Vector Laboratory, Burlingame, CA, USA) according to the manufacturer’s protocol and counterstained with hematoxylin solution Gill no. 3 (Sigma-Aldrich).

## Results

### Analysis of differential gene expression

**Figure 2:**
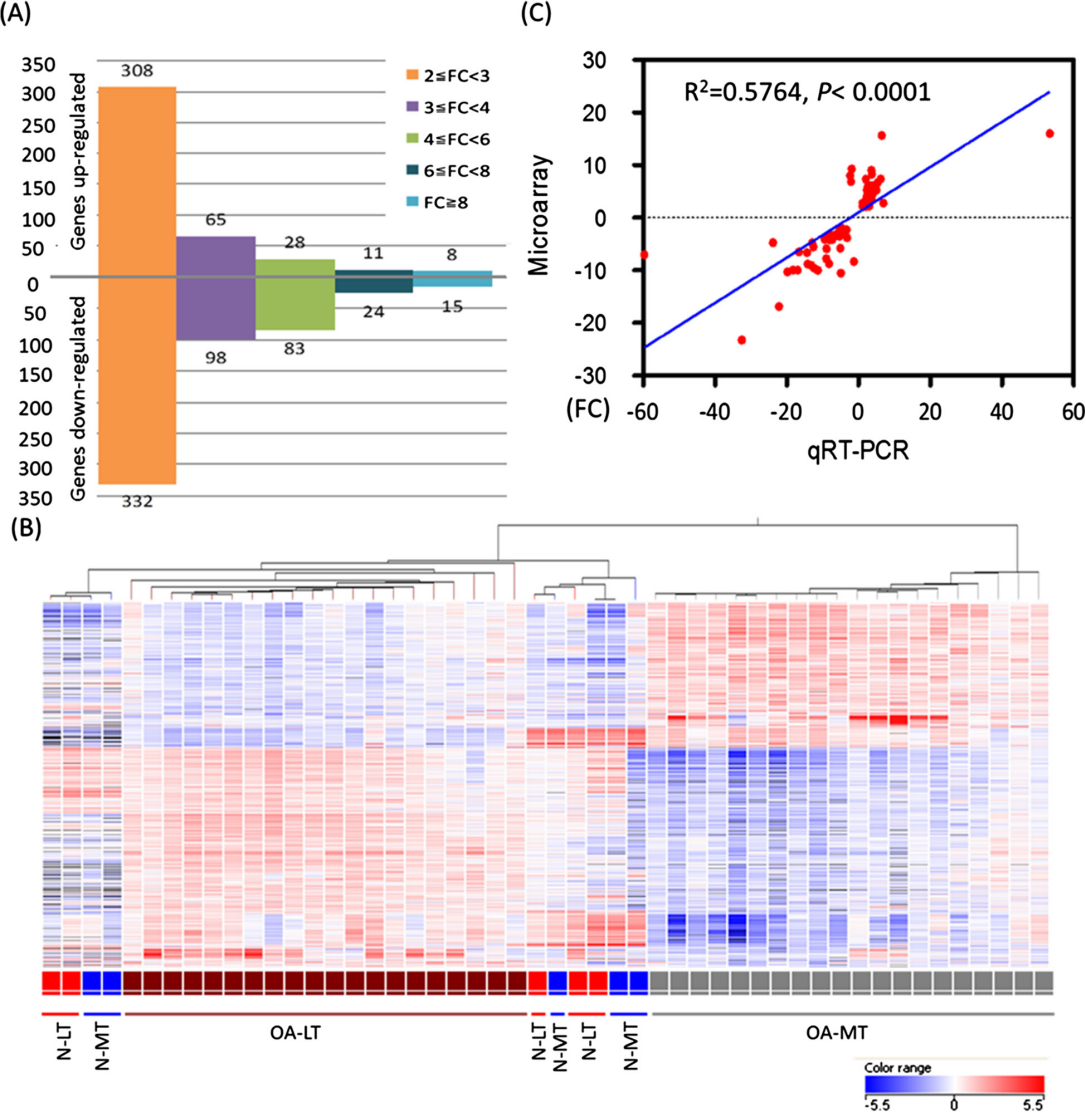
**Microarray analyses of gene expression of osteoarthritis and non-osteoarthritis of subchondral bone and qRT-PCR validation in osteoarthritis knee subchondral bone. (A)** Differentially expressed genes were classified based on their expression levels with a minimum of twofold, threefold, fourfold, sixfold, and eightfold changes. **(B)** Unsupervised hierarchical clustering of osteoarthritis (OA) and non-OA samples was performed for genes whose differential expression exceeded twofold (972 genes). Distances between samples were detected with a Euclidean algorithm and clustered with a centroid linkage method. The OA medial tibial (MT) samples (gray) were clustered together and clearly separated from the OA lateral tibial (LT) (brown) and non-OA samples. Non-OA LT samples (N-LT, red) and non-OA MT samples (N-MT, blue) were clustered together with OA LT samples. **(C)** Eighty-five genes (plus *GAPDH*) were selected for quantitative reverse-transcription polymerase chain reaction (qRT-PCR) validation on a separate group of nine OA subchondral bone specimens. The results of qRT-PCR were strongly consistent with those of microarray analysis (*R*^2^ = 0.5764, *P* <0.0001). FC, fold-change.

For the microarray analysis, 972 differentially expressed genes with ≥2 fold-changes between OA MT and OA LT regions were identified; 420 of these genes were upregulated and 552 were downregulated in OA MT compared with OA LT (Additional file 4). In total, 640 genes were differentially expressed with greater than twofold expression level changes (308 upregulated and 332 downregulated), 163 genes with changes greater than threefold (65 upregulated and 98 downregulated), 111 genes with changes greater than fourfold (28 upregulated and 83 downregulated), 35 genes with changes greater than sixfold (11 upregulated and 24 downregulated), and 23 genes with changes greater than eightfold (eight upregulated and 15 downregulated) (Figure 2A). A graphical depiction of microarray gene expression results across the entire extent of the tibial subchondral bone can be seen in Additional file 4.

Unsupervised hierarchical clustering was performed on the differentially expressed genes to identify the groups or clusters of genes based on similarities between the expression profiles of the samples. On the basis of gene expression profiles, OA LT and OA MT samples were grouped into two distinct clusters. However, the pattern for LT and MT samples from non-OA donors was mixed and did not cluster by sample site; the clustering of all non-OA samples was more akin to the OA LT samples than to the OA MT samples (Figure 2B).

### Quantitative reverse-transcription polymerase chain reaction validation

Nine joint tissue samples were available for validation of microarray results; qRT-PCR analysis of LT and MT subchondral bone regions of these samples was performed on 86 selected genes with *GAPDH* as endogenous control. Based on the results of histological evaluation and micro-computed tomography scanning, the subchondral bone samples selected from the OA MT regions displayed characteristics of severe stages of OA; whereas the OA LT regions displayed characteristics of relatively normal joints. The mean expression pattern of the LT region was compared with the mean expression pattern of MT regions. By qRT-PCR, 77 of 85 genes showed a statistically significant difference between regions (Additional file 5). Expression by qRT-PCR matched the pattern (upregulation or downregulation) of expression observed by microarray, and the two datasets were strongly correlated (*R*^2^ = 0.5764, *P* <0001; Figure 2C). The most differentially upregulated genes in OA MT regions were *IL11*, *POSTN*, *ASPN*, *COL6A3*, *TUBB3*, *COL3A1*, *OGN*, *DIO2*, *PPEF1*, and *TNFSF11*. The most differentially downregulated genes in OA MT regions were *LEP*, *APOB*, *ADH1B*, *CCL8*, *TUSC5*, *KLB*, *NPR3*, *PLIN1*, *PCK1*, and *MYO16.* All of these genes were validated by qRT-PCR except for *ADH1B* and *MYO16*, because these two genes were not available on the microfluidic cards used for qRT-PCR (Table 2).

**Table 2:** Top ten upregulated and downregulated genes comparing lateral tibial bone (below intact cartilage) with medial tibial bone (below lesioned cartilage)

				**Fold-change (MT vs. LT)**		
**Accession number**	**Gene symbol**	**Gene name**	***P *** **value**^**a**^	**OA (microarray)**	**Non-OA (microarray)**	**OA (qRT-PCR)**
Genes with increased expression in medial tibial bone						
NM_000641	*IL11*	Interleukin-11	1.32 × 10^–14^	16.02	1.12	110.31*
NM_006475	*POSTN*	Periostin, osteoblast specific factor	1.48 × 10^–10^	15.65	1.55	9.68*
NM_017680	*ASPN*	Asporin	2.82 × 10^–9^	9.04	2.93	6.34*
NM_057165	*COL6A3*	Collagen, type VI, alpha 3	9.65 × 10^–10^	8.27	1.33	5.11*
NM_006086	*TUBB3*	Tubulin, beta 3	6.98 × 10^–15^	7.36	1.11	6.28*
NM_000090	*COL3A1*	Collagen, type III, alpha 1	5.33 × 10^–16^	7.33	1.32	6.45*
NM_033014	*OGN*	Osteoglycin	7.24 × 10^–10^	6.69	1.70	9.33*
NM_013989	*DIO2*	Deiodinase, iodothyronine, type II	1.58 × 10^–11^	6.20	1.31	5.74*
NM_006240	*PPEF1*	Protein phosphatase, EF-hand calcium binding domain 1	5.31 × 10^–13^	6.14	1.97	4.51*
NM_003701	*TNFSF11*	Tumor necrosis factor (ligand) superfamily, member 11	1.21 × 10^–11^	5.90	1.07	2.64*
Genes with decreased expression in medial tibial bone						
NM_000230	*LEP*	Leptin	4.00 × 10^–10^	−23.27	−2.05	−18.99*
NM_000384	*APOB*	Apolipoprotein B	3.31 × 10^–10^	−16.88	−2.12	−10.20*
NM_000668	*ADH1B*	Alcohol dehydrogenase 1B (class I), beta polypeptide	2.27 × 10^–7^	−12.23	−1.95	NA
NM_005623	*CCL8*	Chemokine (C–C motif) ligand 8	8.17 × 10^–9^	−10.50	−1.59	−6.00*
NM_172367	*TUSC5*	Tumor suppressor candidate 5	4.22 × 10^–10^	−10.25	−1.89	−8.19*
NM_175737	*KLB*	Klotho beta	2.24 × 10^–8^	−10.00	−1.63	−6.09*
NM_001204375	*NPR3*	Natriuretic peptide receptor C	1.36 × 10^–10^	−9.95	−1.38	−7.81*
NM_002666	*PLIN1*	Perilipin 1	2.98 × 10^–8^	−9.60	−1.81	−5.88*
NM_002591	*PCK1*	Phosphoenolpyruvate carboxykinase 1	1.03 × 10^–9^	−9.01	−1.87	−5.44*
NM_001198950	*MYO16*	Myosin XVI	5.81 × 10^–7^	−8.84	−1.93	NA

LT, lateral tibial; MT, medial tibial; NA, not analyzed; OA, osteoarthritis. ^a^Adjusted *P* value for microarray corrected for multiple testing by the Benjamini–Hochberg method. *P <0.001 for quantitative reverse-transcription polymerase chain reaction (qRT-PCR), comparing OA MT versus OA LT. *P* values were determined by two-tailed unpaired Student’s *t* test.

### Functional and pathway analysis

**Figure 3:**
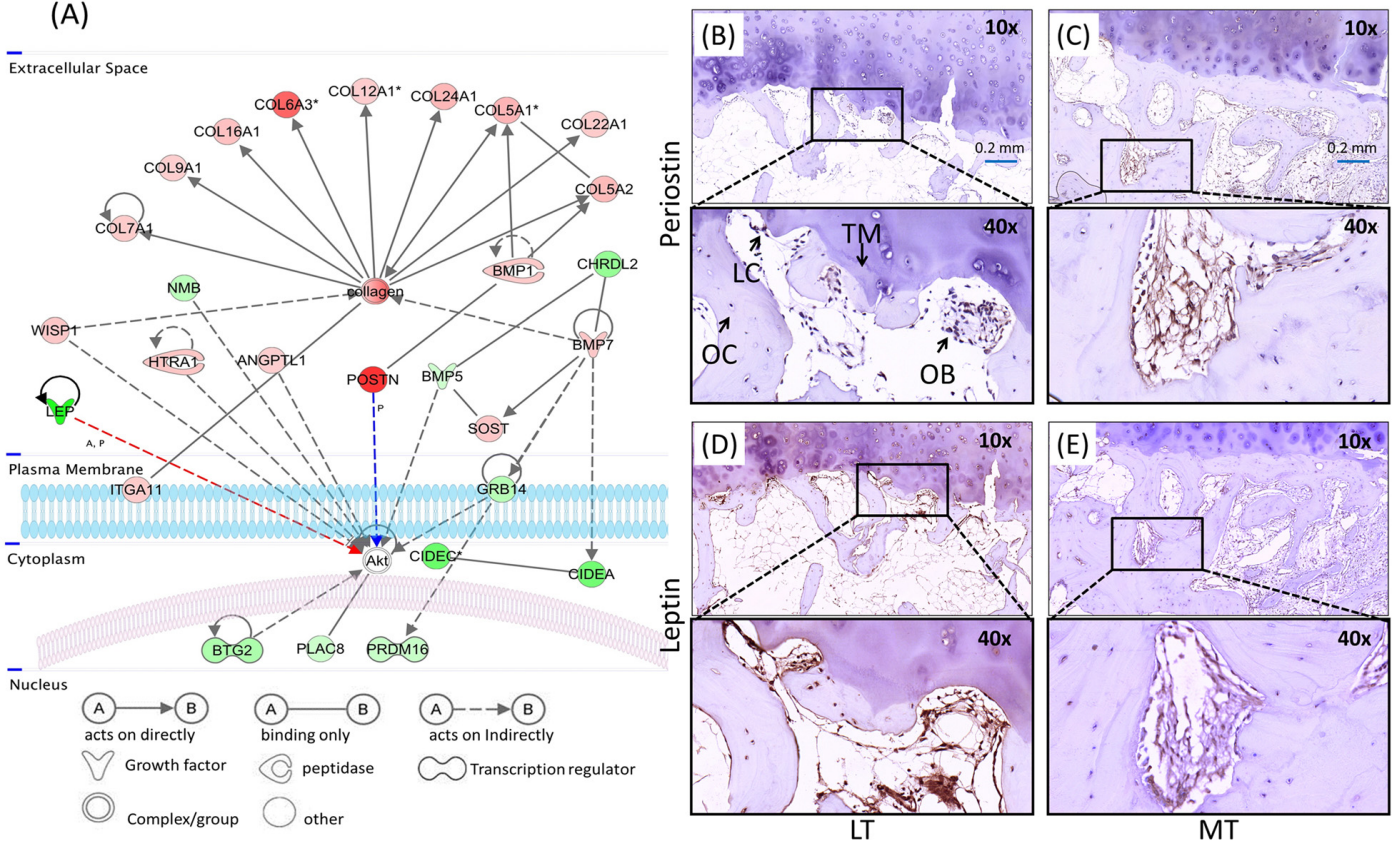
**Prediction of an interactive network of genes and validation of protein expression in osteoarthritis subchondral bone. (A)** Network analysis was performed on differentially expressed genes in the osteoarthritis (OA) samples by ingenuity pathway analysis (IPA). Relationship labels: A, activated; P, phosphorylation/dephosphorylation. **(B)**, **(C)**, **(D)**, **(E)** Antibodies against proteins Periostin and Leptin corresponding to genes *POSTN* and *LEP* were used to detect protein expression (dark brown staining) by immunohistochemistry of OA subchondral bone. Highly expressed, Periostin could be found in osteoblasts (OB) under the tidemark (TM), in osteocytes (OC) and in lining cells (LC) in the OA medial tibial (MT) regions, but not in the OA lateral tibial (LT) regions. Leptin could barely be detected in the OA MT regions, but was strongly expressed in the OA LT regions. All sections were counterstained light blue with hematoxylin. Scale bar = 0.2 mm.

IPA of gene expression profiles revealed 279 functional networks (canonical pathways) (Additional file 6) that were significantly associated with the differentially expressed genes. The predominant associated networks and biological functions were related to lipid metabolism and mineral metabolism (Additional files 7 and 8), connective tissue disorders, cellular growth and proliferation, connective tissue development and function (Additional file 9). Most of these pathways were associated with OA pathogenesis such as bone remodeling, adiposity, connective tissue, and cell activity and death (Table 3). Interestingly, on performing pathway analysis with the most upregulated and downregulated genes, a novel signaling network in OA was identified; this network was generally characterized by upregulation of bone mineralization or collagen-associated genes, such as *COL3A1* (upregulated 7.33-fold, ranking sixth in the top upregulated gene list), *COL6A3* (upregulated 8.27-fold, ranking fourth in the top upregulated gene list), *BMP1*, *BMP7*, *POSTN* (upregulated 15.65-fold, ranking second in the top upregulated gene list), *WISP1*, *HTRA1*, *SOST*, and *ITGA11*, and the downregulation of genes associated with cellular metabolism, proliferation or differentiation (*NMB*, *LEP* (downregulated 23.27-fold, ranking first in the top downregulated gene list), *CHRDL2*, *GRB14*, *CIDEC*, *CIDEA*, *BTG2*, *PLAC8*, and *PRDM16*). Additional differentially expressed genes in this network included *ANGPTL1* (upregulated 2.16-fold), which is involved in angiogenesis. Both *POSTN* and *LEP* may affect the phosphorylation of *AKT* signaling (Figure 3A).

**Table 3:** Genes representative of gene clusters associated with osteoarthritis

**Functional annotation (number of genes, *P *** **value)**^**a**^	**Representative genes**				**Fold-change (MT vs. LT)**		
	**Accession number**	**Gene symbol**	**Gene name**	***P *** **value**^**b**^	**OA (microarray)**	**Non-OA (microarray)**	**OA (qRT-PCR)**
Abnormal morphology of bone	NM_004370	*COL12A1*	Collagen, type XII, alpha 1	6.55 × 10^–08^	2.26	1.01	1.76
(42 genes, *P* = 1.5 × 10^–6^)	NM_000594	*TNF*	Tumor necrosis factor	1.17 × 10^–08^	−3.17	−1.45	−3.42*
Abnormal morphology of collagen fibrils	NM_000093	*COL5A1*	Collagen, type V, alpha 1	2.63 × 10^–08^	2.90	1.35	6.40*
(7 genes, *P* = 1.68 × 10^–6^)	NM_000393	*COL5A2*	Collagen, type V, alpha 2	2.34 × 10^–10^	3.12	1.40	3.18
Adiposity (24 genes, *P* <10^–7^)	NM_003394	*WNT10B*	Wingless-type MMTV integration site Family, member 10B	9.76 × 10^–09^	2.05	−1.08	NA
	NM_001279	*CIDEA*	Cell death-inducing DFFA-like effector a	2.74 × 10^–09^	−6.26	−1.71	NA
Bone mineral density	NM_025237	*SOST*	Sclerostin	2.59 × 10^–04^	2.51	1.44	4.77*
(21 genes, P = 1.1 × 10^–5^)	NM_005357	*LIPE*	Lipase, hormone-sensitive	4.46 × 10^–09^	−8.71	−1.73	−4.21*
Angiogenesis	NM_004673	*ANGPTL1*	Angiopoietin-like 1	2.55 × 10^–06^	2.16	1.02	NA
(69 genes, *P* <10^–7^)	NM_002982	*CCL2*	Chemokine (C–C motif) ligand 2	3.08 × 10^–05^	−3.76	−1.31	−4.87*
Osteoarthritis	NM_001851	*COL9A1*	Collagen, type IX, alpha 1	1.42 × 10^–06^	2.19	−1.05	2.35
(23 genes, *P* <10^–7^)	NM_001463	*FRZB*	Frizzled-related protein	1.36 × 10^–10^	−3.69	1.38	−7.72*
Proliferation of cells	NM_001004439	*ITGA11*	integrin, alpha 11	4.05 × 10^–10^	2.31	−1.03	NA
(303 genes, *P* <10^–7^)	NM_021077	*NMB*	Neuromedin B	1.14 × 10^–06^	−3.00	−1.36	NA
	NM_004490	*GRB14*	Growth factor receptor-bound protein 14	3.38 × 10^–12^	−3.20	−1.18	NA
	NM_006763	*BTG2*	BTG family, member 2	9.11 × 10^–08^	−3.67	−1.31	NA
Quantity of connective tissue	NM_003474	*ADAM12*	ADAM metallopeptidase domain 12	1.86 × 10^–10^	2.30	1.10	1.93
(48 genes, *P* <10^–7^)	NM_022094	*CIDEC*	Cell death-inducing DFFA-like effector c	2.08 × 10^–07^	−6.09	−1.63	NA
Differentiation of cells	NM_054034	*FN1*	Fibronectin 1	1.44 × 10^–07^	2.19	1.38	2.10*
(175 genes, *P* <10^–7^)	NM_016619	*PLAC8*	Placenta-specific 8	1.01 × 10^–08^	−2.28	1.14	NA
Cell movement	NM_000094	*COL7A1*	Collagen, type VII, alpha 1	2.25 × 10^–09^	2.28	−1.00	3.81*
(176 genes, *P* <10^–7^)	NM_012193	*FZD4*	Frizzled family receptor 4	3.62 × 10^–10^	−3.02	−1.37	4.93*
Necrosis	NM_080838	*WISP1*	WNT1 inducible signaling pathway protein 1	3.79 × 10^–08^	2.50	1.09	NA
(218 genes, *P* <10^–7^)	NM_004797	*ADIPOQ*	Adiponectin, C1Q and collagen domain containing	1.71 × 10^–07^	−6.63	−1.69	10.51*
Mineralization of cells	NM_001719	*BMP7*	Bone morphogenetic protein 7	1.65 × 10^–09^	2.50	1.34	2.26**
(14 genes, *P* = 3 × 10^–6^)	NM_015424	*CHRDL2*	Chordin-like 2	1.40 × 10^–04^	−4.04	1.38	−15.71*

LT, lateral tibial; MT, medial tibial; NA, not analyzed; OA, osteoarthritis. ^a^Number of genes associated with the particular ingenuity pathway and differentially expressed in MT versus LT regions of OA samples by microarray analysis; *P* value calculated by Fisher’s exact test. ^b^Adjusted *P* value for microarray corrected for multiple testing by the Benjamini–Hochberg method. *P <0.001 and **P <0.05 for quantitative reverse-transcription polymerase chain reaction (qRT-PCR); *P* <0.05 was considered significant comparing OA MT versus OA LT. *P* values determined by two-tailed unpaired Student’s *t* test.

### Validation of protein expression in subchondral bone specimens

The regions of interest of subchondral bone samples ground for the gene expression study were about 5 mm deep under the calcified cartilage, which included the subchondral bone plate and subarticular spongiosa. These regions have a very heterogeneous complement of cells, including osteoblasts, osteoclasts, osteocytes and bone marrow cells. Although the specific cell types contributing to the changes in gene expression cannot easily be confirmed, all of the cell types in the subchondral bone would be expected to contribute to the subchondral bone gene expression profile. To obtain further independent validation of the microarray results, immunohistochemical staining was performed to investigate the protein expression of proteins Periostin and Leptin corresponding to genes *POSTN* and *LEP* in human OA and non-OA tissue sections from LT and MT regions. The highly expressed Periostin could be found in osteoblasts beneath the tidemark, in osteocytes, and in lining cells of the OA MT regions, but not OA LT regions (Figure 3B,C). Protein expression of Leptin was low in OA MT regions, but strong in OA LT regions (Figure 3D,E). By immunohistochemical analysis, the proteins Periostin and Leptin were not differentially expressed in the MT and LT regions of non-OA samples (Additional file 10).

## Discussion

To the best of our knowledge, this represents the first comprehensive whole genome expression profiling of non-OA and OA subchondral bone. The validity of the microarray results was confirmed by qRT-PCR for a selected subset of genes. Based on analysis by IPA, we identified biological functions implicated in the pathogenesis of OA in bone. Many of the functional networks identified in this study were also associated with processes in connective tissue development and function.

In our previous study [15], we evaluated the expression of 61 OA genes and correlated the expression patterns with the bone morphometric measurement (bone volume). The expression of a total of 45 of 61 genes in the subchondral bone was significantly correlated with the alteration of bone structure including *ADAMTS1*, *ASPN*, *BMP6*, *BMPER*, *CCL2*, *CCL8*, *COL5A1*, *COL6A3*, *COL7A1*, *COL16A1*, *FRZB*, *GDF10*, *MMP3*, *OGN*, *OMD*, *POSTN*, *PTGES*, *TNFSF11*, *WNT1* and others, including the ratio of *OPG* (also known as *TNFRSF11B*) to *RANKL* (also known as *TNFSF11*). *RANKL* is primarily produced from osteocytes, osteoblasts and/or marrow stromal cells and is a key factor for osteoclast differentiation and activation. *RANKL* binds to *OPG*, also known as osteoclastogenesis inhibitory factor, and interacts with the receptor (RANK) for *RANKL*, to modulate the level of osteoclast activity and regulate bone homeostasis in response to various endocrine, paracrine, cytokine and mechanical stresses [17,18]. As shown in Additional file 11, significant correlations were observed for OPG/RANKL and the bone parameters including percent bone volume, bone structure (structure model index), trabecular thickness and trabecular space. These data confirmed our hypothesis that investigations of subchondral bone gene expression changes could provide clues to the pathogenic mechanisms of OA and inform development of new diagnostic markers and therapeutic targets. We focused on the outer LT and central MT regions for the purposes of comprehensive microarray analyses in the current study. The characteristics of the central MT region generally included cartilage matrix loss, cyst formation within cartilage matrix, denuded sclerotic bone, and thickened subchondral bone plate, consistent with a bone sclerosis phenotype [19], whereas the outer LT region was relatively normal. Many of the most significantly regulated genes identified by this microarray study have documented roles in the pathogenesis of OA, arthritis or bone formation including seven of the top 10 upregulated genes (*POSTN*, *ASPN*, *COL6A3*, *COL3A1*, *OGN*, *DIO2*, *TNFSF11*) and two of the top 10 downregulated genes (*LEP*, *APOB*) [20-27]. Through IPA we could also identify the involvement of a lipid metabolism protein network and a mineral metabolism protein network that involved 29 and 22 differentially expressed genes respectively; these networks included *PLINE*, *LIPE*, *DGAT2*, *ADRB1*, *NPY1R*, *HCAR3* and *P2RRY14* that changed −9.6-fold, –8.7-fold, –7.8-fold, –6.2-fold, –7.4-fold, –5.1-fold, and −5.1-fold respectively comparing OA MT with OA LT (Additional files 7 and 8). These functional networks indicated that the bone cells in the OA MT regions were characterized by a condition of low energy production that included a low rate of mineral metabolism. These results are compatible with a low rate of bone remodeling in MT regions.

Increased bone remodeling is an important predictor of OA progression [8] and is characteristic of early stages of OA including resorption of bone and increased porosity in the subchondral bone region [28]. Given that OA MT regions of interest could be defined as representing late stages of OA, we were interested to determine whether OA LT regions of interest could be described as representing early stages of OA. The OARSI histopathological scores of all OA LT samples were <6, representing a relatively intact overlying cartilage matrix with minimal superficial fibrillation not too dissimilar from normal. However, in contrast to normal samples, the bone structural parameters of the OA LT regions had a lower bone volume, thinning of the subchondral bone plate, increased porosity in the subchondral region and reduced bone density (bone density data not shown) suggestive of early OA. Another indicator of early OA, as demonstrated by animal models of OA and in OA patients, is increased bone remodeling characterized by a decreased expression ratio of *OPG* to *RANKL*[29,30]. In contrast to non-OA tissues, a reduced *OPG to RANKL* ratio was identified in OA LT regions compared with non-OA LT regions (*OPG* lacked significant change, but *RANKL* was upregulated 1.63-fold, one-tailed unpaired Student *t* test *P* = 0.048), consistent with early OA. Thus, based on the bone parameters and *OPG* to *RANKL* ratios, comparing LT with non-OA we concluded that the bone gene expression patterns of OA LT regions were consistent with an early-stage OA phenotype.

According to a hypothetical model of OA pathogenesis proposed by Burr and Gallant [6], joint loading increases bone remodeling in the early stage of OA, and a bone–cartilage crosstalk may occur via diffusion of small molecules or increased vascularization at the deep layers of cartilage that could interfere with the normal collagen network. These changes would lead to a loss of aggrecan, increasing inflammation, and result in bone sclerosis at late stages of OA. Several of the genes identified in this study fit nicely into this hypothetical model. For instance, upregulation of *SOST* was identified (2.51-fold); SOST is an inhibitor of Wnt signaling that responds to mechanical loading in the proximal rat tibia and is associated with bone mass changes [31]. In addition, several genes relevant to cell differentiation and activity of osteoblasts, osteoclasts and osteocytes were identified in both a mouse load-regulated gene expression model [32] and in this human study; examples included ASPN (regulator of osteoblast collagen mineralization), WNT5A (agonist of WNT signaling pathway in osteoblasts and osteocytes) and VCAN (regulating transforming growth factor beta expression in osteoclasts). These findings suggest that mechanical loading plays an important role in structural changes of human subchondral bone, initiates bone remodeling, and contributes to the pathogenesis of OA. Moreover, our results could provide a possible novel molecular mechanism to explain changes in bone remodeling during OA development based on an AKT-regulated network. AKT is a serine/threonine-specific protein kinase that plays a pivotal role in many cellular processes such as metabolism, apoptosis, cell proliferation, transcription, cell migration and extracellular matrix alternation [33]. In an early-stage of OA, overexpressed LEP may increase phosphorylation of AKT [34], which will trigger the downstream signaling pathway to increase bone remodeling. In a late stage of OA, upregulated POSTN may inhibit phosphorylation of AKT [35] and will decrease the cellular metabolism, cell proliferation, and differentiation; the rate of bone remodeling would thereby decline.

One of the limitations of this study was that the subchondral bone samples were obtained from OA joints at end-stage disease due to the difficulty of obtaining joints with early stages of OA from humans. However, comparing our results with the gene expression profiles of subchondral bone in an early OA experimental mouse model [12], their reported fold-changes in SB gene expression of ASPN, CCL2, COL3A1, COL5A1, POSTN and TNFSF11 at the initial stages of OA were similar to those of our study. This result strongly supports the credibility of this study. Another limitation was the difficulty distinguishing whether differentially expressed genes reflected OA progression in cartilage, differences in innate bone structure, or were driven by changes in mechanical loading. In our currently study, significant correlation between overlying cartilage integrity and underlying subchondral bone structural parameters could be identified (Additional file 12), suggesting that differential gene expression across the subchondral bone is due, at least in part, to differences in cartilage integrity of the LT and MT regions in OA. Microarray analysis of additional non-OA control samples could help to exclude differentially expressed genes driven by innate bone structure. However, to exclude genes driven by mechanical loading, microarray analysis of lateral compartment dominant OA is necessary.

The site of initiation of OA, at bone or cartilage, has been disputed for decades. Numerous clinical and experimental studies have confirmed that increased bone volume and changes in bone quality of the tibial subchondral bone of the knee are related to loss of cartilage integrity [7,8,36-38]. Goldring and Goldring have pointed out that subchondral bone responds more rapidly to adverse loading and events than cartilage [5]. Moreover, the integrity of subchondral bone predicts later worsening of radiographic osteoarthritis progression [8]. As summarized by Martel-Pelletier and Pelletier, an abnormal rate of bone remodeling, incomplete bone mineralization and increased osteoid collagen matrix will result in hypomineralization of subchondral bone and deterioration of cartilage as manifested by knee OA progression [39]. These data would suggest that drugs designed to target both the bone and cartilage compartments to optimize bone remodeling rates and inhibit cartilage loss in the early stages of OA could inhibit progression of disease. The recent success of strontium ranelate as a disease-modifying agent for OA provides the first clear evidence that this hypothesis has been confirmed [40]. Some other bone-targeting agents have been assessed as OA therapeutics, including bisphosphonates [41,42], calcitonin [43] and vitamin D [44]. These bone-acting agents are unlikely to be effective in late stages of OA, when remodeling is already suppressed. The development of diagnostic biomarkers to detect progressive changes in early stages of OA is therefore important. The genes highly regulated in early stages of OA in our microarray data could provide potential diagnostic biomarkers or therapeutic targets for OA.

## Conclusion

Our microarray results were obtained by examining early and late stages of OA and non-OA samples. Most of the differentially expressed genes in bone are involved in cartilage and bone development, OA pathogenesis and bone remodeling in the early and late stages of OA. This direct assessment of gene expression profiling in OA subchondral bone provides a comprehensive understanding of disease pathology that could further the development of new OA biomarkers and therapeutic strategies.

## Abbreviations

IPA: Ingenuity pathway analysis; LT: Lateral tibial; MT: Medial tibial; OA: Osteoarthritis; OARSI: Histological scoring system to grade severity of osteoarthritis; qRT-PCR: Quantitative reverse-transcription polymerase chain reaction.

## Competing interests

The authors declare that they have no competing interests.

## Authors’ contributions

C-HC carried out the RNA isolation, micro-computed tomography, qRT-PCR, microarray data analysis, statistical analysis, histology and immunohistochemistry experiments, and drafted the manuscript. C-CW, C-HL, H-PC, I-WS, J-HC, L-SL and S-YK participated in RNA isolation and sample collection. J-YW, Y-TC, VBK, and MTML conceived of the study, participated in its design and coordination, and helped to draft the manuscript. MTML had full access to all of the data in the study and takes responsibility for the integrity of the data and the accuracy of the data analysis. All authors read and approved the final version to be published.

## Supplementary Material

Additional file 1 Shows sites of analysis and regions for RNA isolation. Click here for file

Additional file 2 Shows self-organizing maps analysis (SOM) of the differentially expressed genes across the entire tibial plateau. Click here for file

Additional file 3 Presents characteristics of the paired and unpaired OA samples used for microarray analysis. Click here for file

Additional file 4 Presents a list of differentially expressed genes. Click here for file

Additional file 5 Presents gene validation by qRT-PCR. Click here for file

Additional file 6 Presents a list of canonical pathways. Click here for file

Additional file 7 Shows a potential network of lipid metabolism in OA. Click here for file

Additional file 8 Shows a potential network of mineral metabolism in OA. Click here for file

Additional file 9 Presents a list of dominant functional networks. Click here for file

Additional file 10 Shows validation of protein expression in human non-OA knee samples. Click here for file

Additional file 11 Presents correlation of OPG/RANKL and structural parameters of the subchondral bone. Click here for file

Additional file 12 Presents correlations between cartilage integrity and the bone parameters. Click here for file
