# Supplementary material for: Genome-wide expression profiles of subchondral bone in osteoarthritis
Source: Arthritis Res Ther. 2013 Nov 15;15(6):R190. doi: 10.1186/ar4380 (PMC3979015; doi:10.1186/ar4380)
Supplement: Additional file 1 — Shows sites of analysis and regions for RNA isolation. [file ar4380-S1.docx]

**Additional file 1.** **Sites of analysis and regions for RNA isolation**

Human osteoarthritic knee tibial plateau; the red boxes indicate regions for mCT and histological analysis at the four regions of interest; the dashed boxes demonstrate regions for RNA isolation: outer lateral tibial plateau (oLT); inner lateral tibial plateau; inner medial tibial plateau; and the central medial tibial plateau devoid of cartilage.
