# Supplementary material for: Genome-wide expression profiles of subchondral bone in osteoarthritis
Source: Arthritis Res Ther. 2013 Nov 15;15(6):R190. doi: 10.1186/ar4380 (PMC3979015; doi:10.1186/ar4380)
Supplement: Additional file 2 — Shows self-organizing maps analysis (SOM) of the differentially expressed genes across the entire tibial plateau. [file ar4380-S2.docx]

**
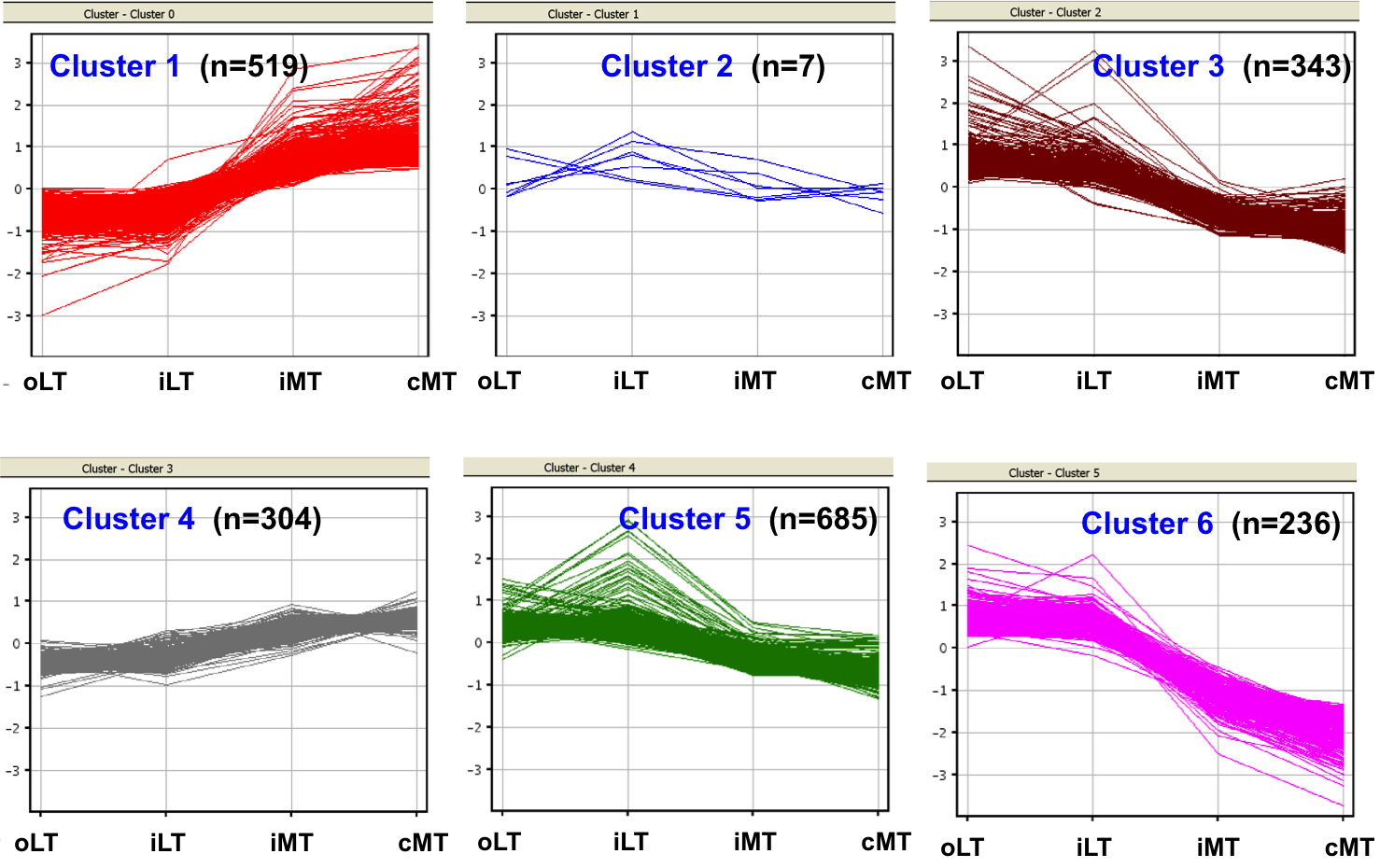
 Additional File 2. Self-organizing maps analysis (SOM) of the differentially expressed genes across the entire tibial plateau.**

2092 differentially expressed genes with greater than 2-fold changes between outer lateral tibial plateau (oLT), inner lateral tibial plateau (iLT), inner medial tibial plateau (iMT), and center medial tibial plateau (cMT) were identified in microarray analysis (data not shown). SOM analysis yielded 6 clusters. Each cluster is characterized by a specific gene expression pattern. Some genes changed dramatically across the entire tibial plateau (clusters 1 and 6). Some genes changed modestly across the entire tibial plateu (clusters 3 and 4) and some genes changed little (clusters 2 and 5). The representative genes in these clusters include the following: *POSTN* (cluster 1), *KLF4* (cluster 2), *FRZB* (cluster 3), *TNFRSF11B* (cluster 4), *GDF10* (Cluster 5), and *LEP* (cluster 6). The gene expression patterns between oLT and iLT samples, and between iMT and cMT samples were similar, but clearly separated between the OA-LT and OA-MT samples. For these reasons, we focused on the comparison between OA-oLT and OA-MT (combination of iMT and cMT) regions for the current study. The numbers in each panel show the number of genes belonging to the corresponding cluster.
