# Supplementary material for: Genome-wide expression profiles of subchondral bone in osteoarthritis
Source: Arthritis Res Ther. 2013 Nov 15;15(6):R190. doi: 10.1186/ar4380 (PMC3979015; doi:10.1186/ar4380)
Supplement: Additional file 3 — Presents characteristics of the paired and unpaired OA samples used for microarray analysis. [file ar4380-S3.docx]

**Additional file 3. Characteristics of the paired and unpaired OA samples used for microarray analysis**

Values are the mean ± SD.

BV/TV = Percent bone volume; SMI = Structure model index

Tb.Th =Trabecular thickness; Tb.N = Trabecular number; Tb.Sp = Trabecular separation
