# Supplementary material for: Genome-wide expression profiles of subchondral bone in osteoarthritis
Source: Arthritis Res Ther. 2013 Nov 15;15(6):R190. doi: 10.1186/ar4380 (PMC3979015; doi:10.1186/ar4380)
Supplement: Additional file 7 — Shows a potential network of lipid metabolism in OA. [file ar4380-S7.docx]

**
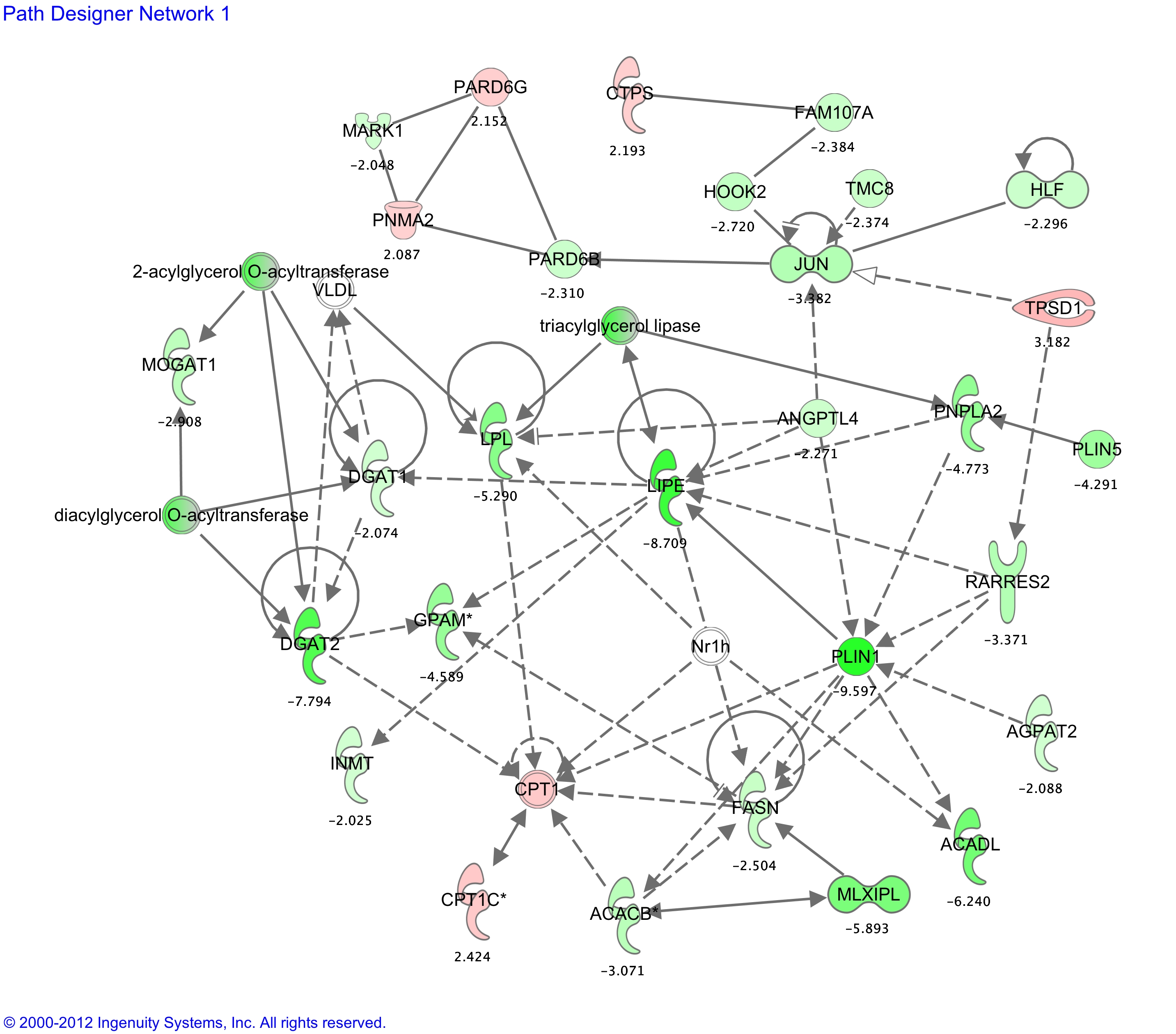
**

**Additional File 7. A potential network of lipid metabolism in OA.**

Based on microarray and IPA analyses, a network of genes related to lipid metabolism was associated with OA. The expression intensity ratios of OA-MT versus OA-LT are indicated below the gene icons. Up-regulated and down-regulated genes are highlighted in red and green respectively with the fold change indicated.
