# Supplementary material for: Genome-wide expression profiles of subchondral bone in osteoarthritis
Source: Arthritis Res Ther. 2013 Nov 15;15(6):R190. doi: 10.1186/ar4380 (PMC3979015; doi:10.1186/ar4380)
Supplement: Additional file 10 — Shows validation of protein expression in human non-OA knee samples. [file ar4380-S10.docx]

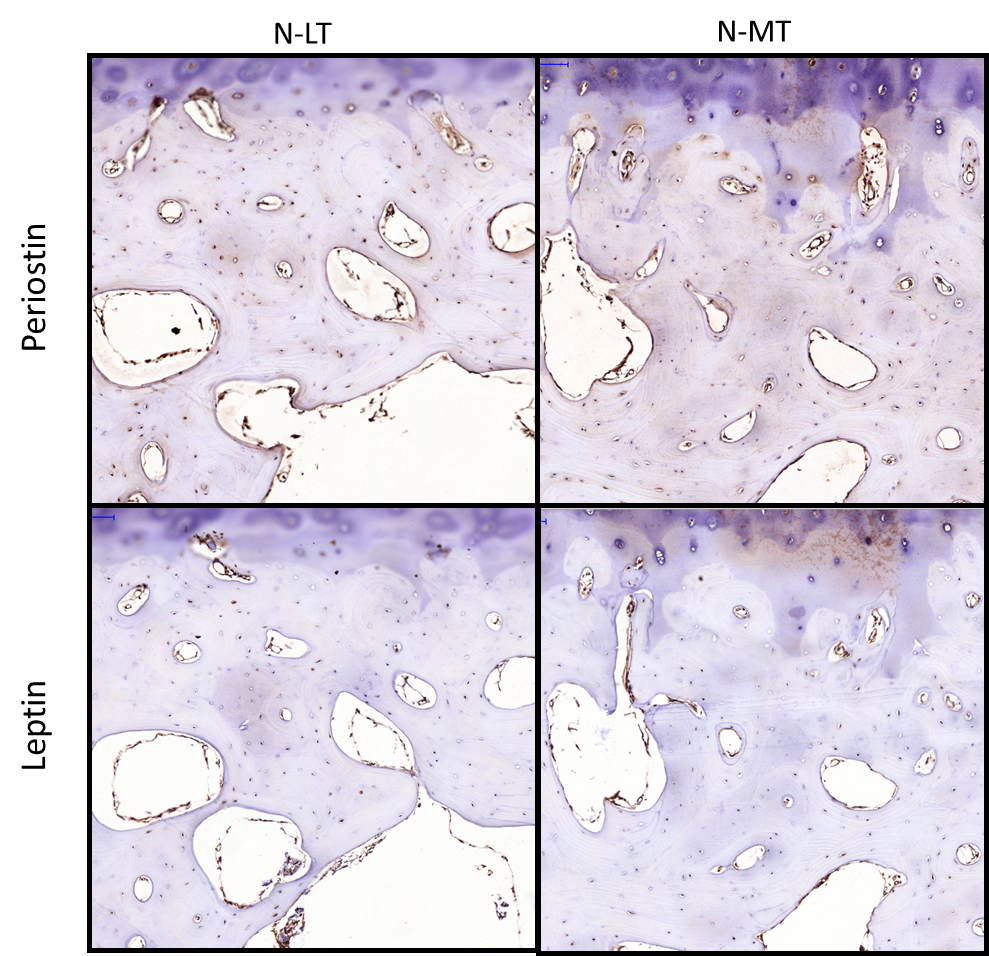


**Additional File 10.** **Validation of protein expression in human non-OA knee samples.**

Antibodies against POSTN, LEP and p-AKT were used to detect protein expression (dark brown staining) by Immunohistochemistry in human non-OA knee subchondral bone. No differences in protein expression were observed between lateral tibial (N-LT) and medial tibial (N-MT) regions.
