# Supplementary material for: Genome-wide expression profiles of subchondral bone in osteoarthritis
Source: Arthritis Res Ther. 2013 Nov 15;15(6):R190. doi: 10.1186/ar4380 (PMC3979015; doi:10.1186/ar4380)
Supplement: Additional file 11 — Presents correlation of OPG/RANKL and structural parameters of the subchondral bone. [file ar4380-S11.docx]

**Additional File-11. Correlation of OPG/RANKL and structural parameters of the subchondral bone.**
