# Supplementary material for: Genome-wide expression profiles of subchondral bone in osteoarthritis
Source: Arthritis Res Ther. 2013 Nov 15;15(6):R190. doi: 10.1186/ar4380 (PMC3979015; doi:10.1186/ar4380)
Supplement: Additional file 12 — Presents correlations between cartilage integrity and the bone parameters. [file ar4380-S12.docx]

**Additional File 12. Correlations between cartilage integrity and the bone parameters.**
